# Comprehensible Predictive Modeling Using Regularized Logistic Regression and Comorbidity Based Features

**DOI:** 10.1371/journal.pone.0144439

**Published:** 2015-12-08

**Authors:** Gregor Stiglic, Petra Povalej Brzan, Nino Fijacko, Fei Wang, Boris Delibasic, Alexandros Kalousis, Zoran Obradovic

**Affiliations:** 1 Faculty of Health Sciences, University of Maribor, Maribor, Slovenia; 2 Faculty of Electrical Engineering and Computer Science, University of Maribor, Maribor, Slovenia; 3 Department of Computer Science and Engineering, University of Connecticut, Storrs, Connecticut, United States of America; 4 Faculty of Organizational Sciences, University of Belgrade, Belgrade, Serbia; 5 Department of Business Informatics, University of Applied Sciences Western Switzerland, Carouge, Switzerland; 6 Computer Science Department, University of Geneva, Geneva, Switzerland; 7 Center for Data Analytics and Biomedical Informatics, Temple University, Philadelphia, Pennsylvania, United States of America; New Jersey Institute of Technology, UNITED STATES

## Abstract

Different studies have demonstrated the importance of comorbidities to better understand the origin and evolution of medical complications. This study focuses on improvement of the predictive model interpretability based on simple logical features representing comorbidities. We use group lasso based feature interaction discovery followed by a post-processing step, where simple logic terms are added. In the final step, we reduce the feature set by applying lasso logistic regression to obtain a compact set of non-zero coefficients that represent a more comprehensible predictive model. The effectiveness of the proposed approach was demonstrated on a pediatric hospital discharge dataset that was used to build a readmission risk estimation model. The evaluation of the proposed method demonstrates a reduction of the initial set of features in a regression model by 72%, with a slight improvement in the Area Under the ROC Curve metric from 0.763 (95% CI: 0.755–0.771) to 0.769 (95% CI: 0.761–0.777). Additionally, our results show improvement in comprehensibility of the final predictive model using simple comorbidity based terms for logistic regression.

## Introduction

In the recent revolution in the healthcare field that focuses on big data driven predictive models that will be able to offer decision support on a personalized level, we still mostly use classification and regression models that were developed decades ago. However, even though some basic methodologies stay the same, we have recently witnessed many adaptations of different predictive methods due to the increasing the number of available samples and especially due to a rapid pace of growth in the number of available features. A large part of such adaptations comes from bioinformatics where the need for adaptations of older predictive models originates from specific dataset characteristics such as a feature dimensionality that is much larger than the number of available examples. Different variants of regression models have been widely used in the biomedical domain to build effective and interpretable predictive models [1]. The rapid increase in the number of features brought some novel adaptations of regression models that now allow us to simultaneously select features and predict the target class or value [2]. Also, adding interactions to the prediction models can provide more information considering the co-occurrence effects of different features, and for complex prediction problems traditional additive models are insufficient [3–5]. However, adding all pairwise interactions in a dataset with *n* features will result in a model with 2^*n*^ terms considering only interactions of second order and will grow exponentially with the number of features. This not only highly increases the computation burden, but also the complexity of the model. For that reasons, most researchers only focus on pairwise interaction models [3, 6].

The interaction discovery method that was originally proposed to solve very high-dimensional problems in bioinformatics was used in this study to pre-filter a set of features and their interactions. This procedure is followed by the introduction of simple logical terms and a post-processing that allows the building of effective and comprehensible predictive models from Electronic Medical Records (EMR) data. The high dimensionality of data in EMR usually originates from binary features representing diagnoses, procedures or drugs using specific diagnostic, procedural and pharmaceutical codes. In general, the interactions corresponding to the features with larger main effects intuitively have more practical effects on the output [7]. For that reason we focus on reliable interactions that have larger main effects.

Information on comorbidities or co-occurrence of multiple diseases has been widely used to improve different algorithms. In this paper, we use the terms comorbidity and interaction interchangeably, which refer to a co-occurrence of two or more diagnoses or clinical conditions. Lappenshaar et al. [8] proposed a novel method to identify interactions between different malignancies including their interpretation based on Bayesian networks. Riano et al. [9] introduced a model for combination of treatments for the management of chronic comorbid patients using a divide-and-conquer approach. The comorbidity of hypertension and chronic heart failure was explored in the evaluation of the proposed approach. However, none of the two mentioned studies used comorbidity data in predictive models. Most methods that employ co-occurrence of features in high-dimensional data have their origins in bioinformatics. A study by Ruczinski et al. [10] presents a regression method that uses Boolean combinations of binary features as new features to improve the regression performance. Although this approach can be used to improve the regression performance by adding interpretable features, the new features often consist of multiple basic features combined in complex representations. Combined new features can be difficult to interpret, especially when multiple such features are combined in a regression equation. Bien et al. [3] and Lim and Hastie [11] proposed two approaches limited to the discovery of regression interaction terms instead of more complex features. Both of them use highly efficient lasso regression [12] that allows them to find a small set of interaction terms contributing to the improvement of the regression performance. Both approaches also satisfy strong hierarchical constraints, i.e. the inclusion of an interaction term also implies the inclusion of its main effects in the regression equation. Therefore, our approach relies on the work of Lim and Hastie that allows for the fast selection of the initial set of interaction terms. As an alternative to regularized regression, one can use other rule-based systems that allow interpretability of results. In this paper, we use boosted decision trees [13], where feature selection is part of the decision tree building process. Although small sets of boosted decision trees can achieve competitive results [14], it is difficult to interpret the obtained models. Even though each boosted decision tree can be transformed to a set of rules for easier interpretation, we have to be aware that each boosted decision tree should be interpreted separately as opposed to simply merging all rules obtained in the process of decision tree boosting.

## Materials and Methods

In the initial step of our approach, we use a *glinternet* R package by Lim and Hastie [11] due to the lower computational complexity of this approach as compared to competing solutions. In this study, we measure the complexity or comprehensibility of the predictive model by the number of regression coefficients. We follow the intuition that if we reduce the number of features (i.e. regression coefficients) in the model we facilitate its interpretation by the domain expert. Our approach consists of three steps:

1. Run group lasso based approach for learning pairwise interactions in a manner that satisfies strong hierarchy to obtain a set of main effects with corresponding interactions. Here, a user defines the number of interactions to be discovered.
2. Use the information on the selected features to introduce additional Boolean logic based features for binary features representing presence of diagnoses on a medical record. The introduction of these additional features allows the model to capture the cases when one diagnosis is present while the other is not (e.g. discerning subgroups of patients with similar diagnosis codes on their records).
3. Run lasso regression to find an optimal set of features introduced in step 2.

Here, we explain the introduction of new features from step 2 for a simple case of a single interaction involving two binary features representing presence of diagnoses *d*_*1*_ and *d*_*2*_.

As a result of step 1, we obtain the following basic regression equation:

$$y=\beta_{0}+\beta_{1}d_{1}+\beta_{2}d_{2}+\beta_{3}d_{1}d_{2}+\varepsilon$$

where y represents the output of the regression function, β is the regression coefficient, and *ε* is the random error term. More generally, for presence of multiple diagnoses the following regression equation is obtained:

$$y=\beta_{0}+\underset{j}{\sum^{\text{​}}}\beta_{j}d_{j}+\frac{1}{2}\underset{j\neq k}{\sum^{\text{​}}}\theta_{jk}d_{j}d_{k}+\varepsilon$$

where j and k represent the number of diagnoses; *θ* is introduced as a coefficient for interaction terms.

Since *glinternet* was used to obtain the above result, the model respects the hierarchy constraints by including the main effects and the interaction term in the equation. At this point, we add two additional logical features to the equation as follows:

$$y=\beta_{0}+\beta_{1}d_{1}+\beta_{2}d_{2}+\beta_{3}d_{1}d_{2}+\beta_{4}d_{1}d_{2}^{c}+\beta_{5}d_{1}^{c}d_{2}+\varepsilon$$

A more general form of the extended equation that includes additional regression coefficients is:

$$y=\beta_{0}+\underset{j}{\sum^{\text{​}}}\beta_{j}d_{j}+\frac{1}{2}\underset{j\neq k}{\sum^{\text{​}}}\theta_{jk}d_{j}d_{k}+\underset{j<k}{\sum^{\text{​}}}\gamma_{jk}d_{j}d_{k}^{c}+\underset{j<k}{\sum^{\text{​}}}\delta_{jk}d_{j}^{c}d_{k}+\varepsilon$$

where $d_{j}d_{k}^{c}$ and $d_{j}^{c}d_{k}$ represent cases where only one of the diagnoses is present and the other is not, *β* represents the coefficients of single diagnoses, while *θ*, *γ* and *δ* represent coefficients for different types of interactions.

Next, we reduce the number of coefficients based on the optimal performance that can be achieved by keeping only the coefficients for features selected by lasso regression at non-zero. This way we can reduce the number of features and improve the comprehensibility of our model. We used the proposed approach on a binary classification problem and therefore a logistic regression was used instead of the more general multivariate regression. Our approach improves regression performance and reduces the number of features in the final model, thus improving the interpretability of the model as demonstrated in the next section. Although we lose the strong hierarchy by introduction of a lasso regression in the third step, we are able to capture relations including both diagnoses by the newly introduced features from step 2 and therefore reduce the need for the strong hierarchy. This is especially true in cases where one or both of the newly introduced coefficients are kept in the final model, with all the rest eliminated.

To compare the results with alternative solutions that still maintain the interpretability of results, we ran additional experiments where we used boosted decision trees, using C5.0 package in R [15, 16], instead of lasso regression in the final step of the proposed approach. We used 10 boosting iterations and kept the rest of the parameters at their default values—i.e. minimal number of samples in leaves was set to 2, confidence factor to 0.25 with global pruning off and early stopping on. The source code for all experiments performed in the paper is available from https://github.com/gregst/plos-one/.

## Results

The empirical evaluation of the proposed method with respect to its predictive performance as well as the complexity of the final model was done on the problem of predicting rehospitalization within 30 days from the date of discharge. Hospital discharge data from California, State Inpatient Databases (SID), Healthcare Cost and Utilization Project (HCUP), Agency for Healthcare Research and Quality [17] was used in all experiments. The SID is a component of the HCUP, a partnership between federal and state governments and industry, tracking all hospital admissions at the individual level. The HCUP data is open and available to all researchers. We used data from January 2009 through December 2011 in the pre-processing phase. In this study, we focus on a specific population of patients from pediatric hospitals that are rarely used in studies predicting readmission, but represent an important group where good results in terms of predictive performance can be achieved [18]. After pre-processing the data from pediatric hospitals (including children up to 10 years of age), we obtained the final dataset containing 61,111 discharge records with 10,675 positive (readmitted within 30 days) and 50,436 negative records.

Features used to perform the classification, included age, sex, length of stay, number of chronic diseases, number of procedures on the record and total charges in USD. An additional set of 213 features representing the presence of most frequent diagnoses was added to an initial set of general information. In case of total charges and length of stay, the log-transformed features were added as well.

To measure the performance of the compared methods, we use the repeated hold-out set evaluation where we randomly split the data into a training set consisting of 2/3 and a test set consisting of the remaining 1/3 of data samples. The hold-out based evaluation was repeated 1000 times to obtain a better insight into the variance of the area under the ROC curve (AUC) and complexity of the predictive model (number of coefficients) that were measured for each run.

In all experiments, we compared the performance of the group lasso interaction discovery method called *glinternet* as proposed by Lim and Hastie [11] to our proposed approach that aims to reduce the complexity of the predictive model and keep the regression terms highly interpretable at the same time. Four experimental runs were conducted where the number of discovered interactions (*NDI*) was set to 5, 10, 15 and 20. The λ parameter that controls the overall strength of the penalty on regression coefficients was tuned on the training set using 5-fold cross-validation to avoid feature selection bias as described by Friedman et al. [19]. The same λ parameter tuning was used in step 1 (glinternet) and step 3 (lasso) of the proposed approach. Two settings were used in the application of lasso (step 3) in our approach. The first one used λ that resulted in the optimal AUC (*OPT*), while the second allowed one standard error deviation from AUC but resulted in a smaller set of selected features (*1SE*).

### Performance and Interpretability

**Fig 1:**
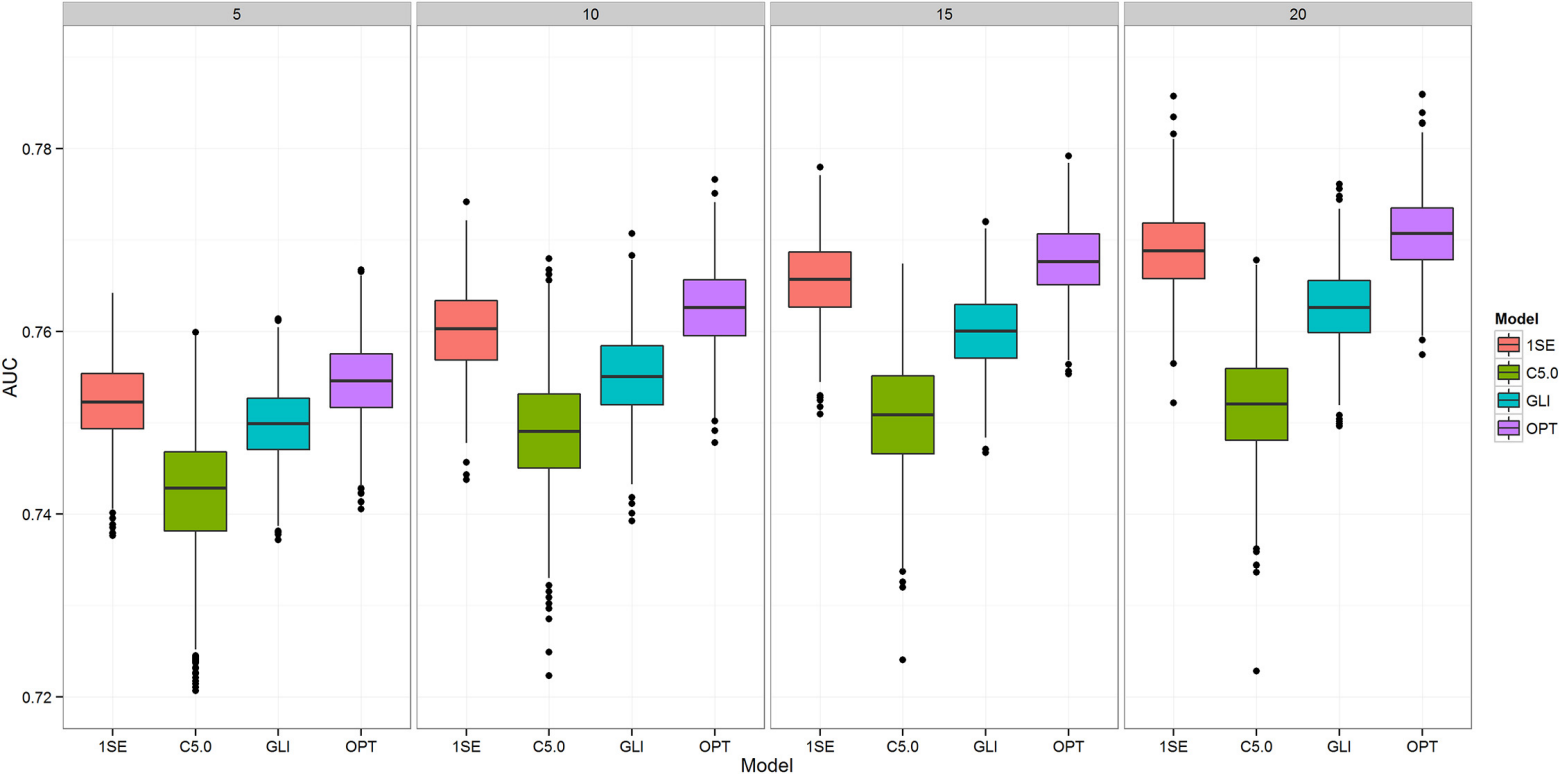
Classification performance of the three observed approaches. Four sets of boxplots represent predictive performance measured in Area under the ROC curve (AUC) for 1-Standard Error (1SE), boosted C5.0 decision trees (C5.0), glinternet (GLI) and a model using optimal lambda (OPT) setting obtained using cross-validation. Each set is obtained for a different setting of “Number of Discovered Interactions” (NDI)–i.e. 5, 10, 15 and 20 interactions.

Fig 1 presents the results for the AUC, where it can be observed that the models produced by OPT and 1SE resulted in an improved AUC compared to *glinternet*. However, the differences in AUC are not significantly different with an average AUC for *glinternet* with 5 interactions at 0.750 (95% CI: 0.742–0.758), OPT at 0.756 (0.747–0.764) and 1SE at 0.754 (0.746–0.763). The best results were obtained with 20 interactions with the average AUC for glinternet 0.763 (0.755–0.771), OPT 0.771 (0.763–0.779) and 1SE 0.769 (0.761–0.777). The only exception to the above results was the performance of the C5.0 decision trees with AUC of 0.742 (0.722–0.755) for 5 interactions and 0.752 (0.741–0.762) for 20 interactions that differs significantly, especially as the number of interactions increases.

**Fig 2:**
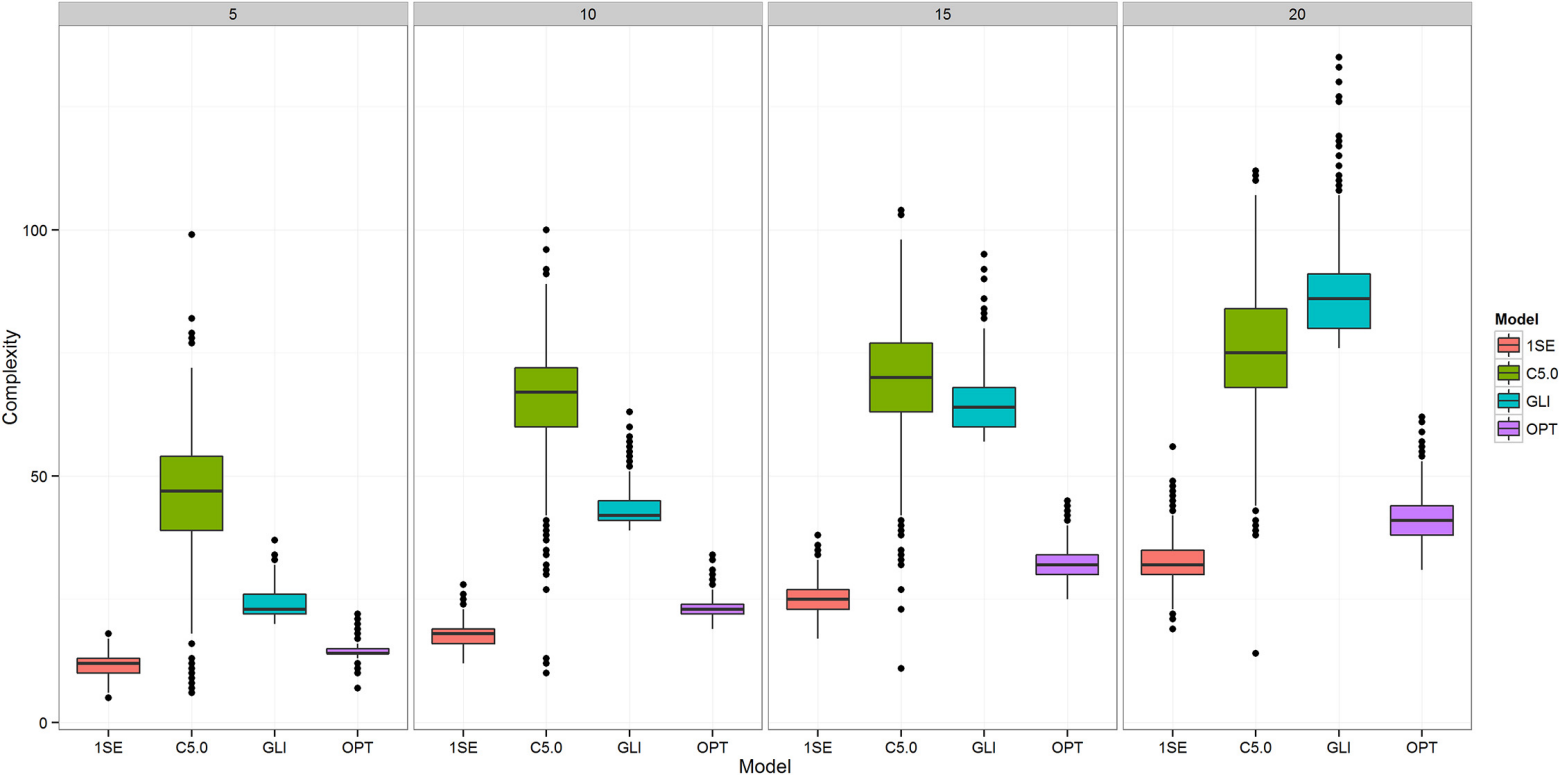
Complexity of the three observed approaches. Comparison of model complexity, measured as number of selected features, for the three compared approaches and four different settings of “Number of Discovered Interactions” (NDI).

Since this study primarily focuses on the reduction of model complexity and consequently improved comprehensibility, one expects significant differences in the number of features selected by the different approaches. Fig 2 confirms our expectations and demonstrates significant differences for OPT and 1SE in comparison to baseline (glinternet) and C5.0 models. In the case of 5 selected interactions we can observe a relatively low number of selected features for all methods with average number of selected features for C5.0 trees at 45.99 (95% CI: 18.00–66.00), *glinternet* at 24.11 (21.00–31.18), OPT at 10.02 (6.00–17.00) and 1SE at 6.41 (5.00–11.00). When comparing the most complex models that performed the best in terms of AUC, we can observe bigger differences. Here C5.0 used 75.95 (51.73–100.00), glinternet used 87.11 (78.00–107.23), OPT 40.43 (32.00–52.00) and 1SE 24.55 (15.00–38.00) features on average.

### Comorbidity Analysis

To compare the results of the proposed OPT and 1SE models, we analyzed different sets of features selected in 1000 experimental runs with emphasis on significant interactions representing comorbidities of interest. As can be seen, especially from Table 1, the simple logic features that were introduced in our approach play an important role in predicting the readmission within 30 days of the hospital discharge.

**Table 1:** Ranked list of the most frequent positive coefficients including comorbidity terms for both proposed approaches.

Rank	Variable	Present in the final set of features (%)	
		OPT	1SE
1	288.00 = 0 AND 204.00 = 1; 288.00—Neutropenia, unspecified; 204.00—Acute lymphoid leukemia, without mention of having achieved remission	100.0	99.9
2	288.00 = 1 AND 204.00 = 0; 288.00—Neutropenia, unspecified; 204.00—Acute lymphoid leukemia, without mention of having achieved remission	100.0	100.0
3	V58.11 = 0 AND 194.0 = 1; V58.11—Encounter for antineoplastic chemotherapy; 194.0—Malignant neoplasm of adrenal gland	100.0	100.0
4	V58.11 = 0 AND 284.1 = 1; V58.11—Encounter for antineoplastic chemotherapy; 284.1—Pancytopenia	100.0	100.0
5	Length of stay (log transformed)	100.0	100.0
6	Number of chronic conditions	100.0	100.0
7	V58.11 = 1 AND 288.00 = 0; V58.11—Encounter for antineoplastic chemotherapy; 288.00—Neutropenia, unspecified	99.8	99.9
8	V58.11 = 0 AND 780.60 = 1; V58.11—Encounter for antineoplastic chemotherapy; 780.60—Fever, unspecified	99.2	93.8
9	V58.11 = 0 AND 780.61 = 1; V58.11—Encounter for antineoplastic chemotherapy; 780.61—Fever presenting with conditions classified elsewhere	99.2	99.3
10	V58.11 = 1 AND 284.1 = 0; V58.11—Encounter for antineoplastic chemotherapy; 284.1 –Pancytopenia	99.0	97.5

One can notice that the two highest ranked features both include ICD9-CM diagnosis codes 288.00 (Neutropenia, unspecified) and 204.00 (Acute lymphoid leukemia, without mention of having achieved remission) and might represent an interesting “switching” pattern. Consequently, this means that for patients that have only one of those two diagnoses the risk of readmission is higher than for patients that do not have any or have both of the two diagnoses. One has to be aware that frequent selection of a variable does not necessarily imply a strong influence on the dependent variable. However, in the above case it can be observed that a feature with both 288.00 and 204.00 was not present, except in a very few cases (0.2%) for the OPT model (S1 File). On the other hand, it is also possible that 204.00 confounds 288.00 or vice-versa. Further experiments would be needed to confirm this.

Additionally, selected comorbidities can also lead to discovery of new knowledge or confirmation of already existing knowledge on comorbidities. For example, an interaction with negative coefficient between Pneumonia (486) and Obstructive sleep apnea (327.23) is mentioned in a recent paper by Bhattacharyya [20] who reported their relation to readmissions in adult population. Our results point at a similar relation in the pediatric population. In our case, presence of 486 and 327.23 can be observed in the two top ranked features with an additional feature where only 486 is present as a frequent negative coefficient (Table 2). For all hospitalizations where 486 = 0 AND 327.23 = 1 or 486 = 1 AND 327.23 = 0, the risk of readmission is reduced, with an additional reduction for the second group that has only 486 present. This means that even in case of both 486 and 327.23 being present the readmission risk will mostly be reduced due to high frequency of 486 in negative coefficients, however this reduction will be smaller than in case of patients where only one of the two diagnoses is present.

**Table 2:** Ranked list of the most frequent negative coefficients including comorbidity terms for both proposed approaches.

Rank	Variable	Present in the final set of features (%)	
		OPT	1SE
1	Number of chronic conditions AND Age	100.0	46.1
2	Number of chronic conditions AND Length of stay (log transformed)	100.0	8.87
3	Number of chronic conditions AND Number of procedures	99.3	19.1
4	486 = 0 AND 327.23 = 1; 486—Pneumonia, organism unspecified; 327.23—Obstructive sleep apnea (adult)(pediatric)	97.8	94.8
5	486—Pneumonia, organism unspecified	82.9	85.3
6	486 = 1 AND 327.23 = 0; 486—Pneumonia, organism unspecified; 327.23—Obstructive sleep apnea (adult)(pediatric)	72.1	51.9
7	486 = 0 AND 382.9 = 1; 486—Pneumonia, organism unspecified; 382.9—Unspecified otitis media	69.7	58.8
8	799.02 –Hypoxemia	57.8	61.3
9	493.00—Extrinsic asthma, unspecified	53.7	55.5
10	382.9—Unspecified otitis media	39.4	45.5

With a closer observation of the results in Tables 1 and 2, one can notice the presence of two numerical features in both tables—i.e. log transformed length of stay (LOS_LOG) and number of chronic conditions (NCHRONIC). As main effects, they represent a positive coefficient in all models, but on the other side, they are also present in all models as a negative interaction coefficient. Practically, this means that for children who are hospitalized for a longer period and have a high number of chronic conditions the hospitalization risk decreases, in contrast to children with lower length of stay and high number of chronic conditions or higher length of stay and lower number of chronic conditions.

In Table 2, we see that the top ranked features of OPT and 1SE differ significantly. By allowing up to one standard deviation larger error in lasso regression *λ* tuning to achieve a smaller more comprehensible model, we are reducing the cardinality of the set of selected features in the 1SE model. It is intuitive that 1SE will set some similar (i.e. highly correlated) features to zero and keep only a few of the similar features. For example, in Table 2 the similarity between the top 3 features can be clearly seen—they all include number of chronic conditions. Since we want to reduce the set of features by employing 1SE, it seems that in most cases only one of the three similar features (or even none in some cases) was chosen. Based on the results from Table 2 we can conclude that interaction with age, length of stay or number of procedures gives very similar results. However, if we allow more features to be selected, each of them can be useful and possibly contribute to a slight improvement in AUC.

**Fig 3:**
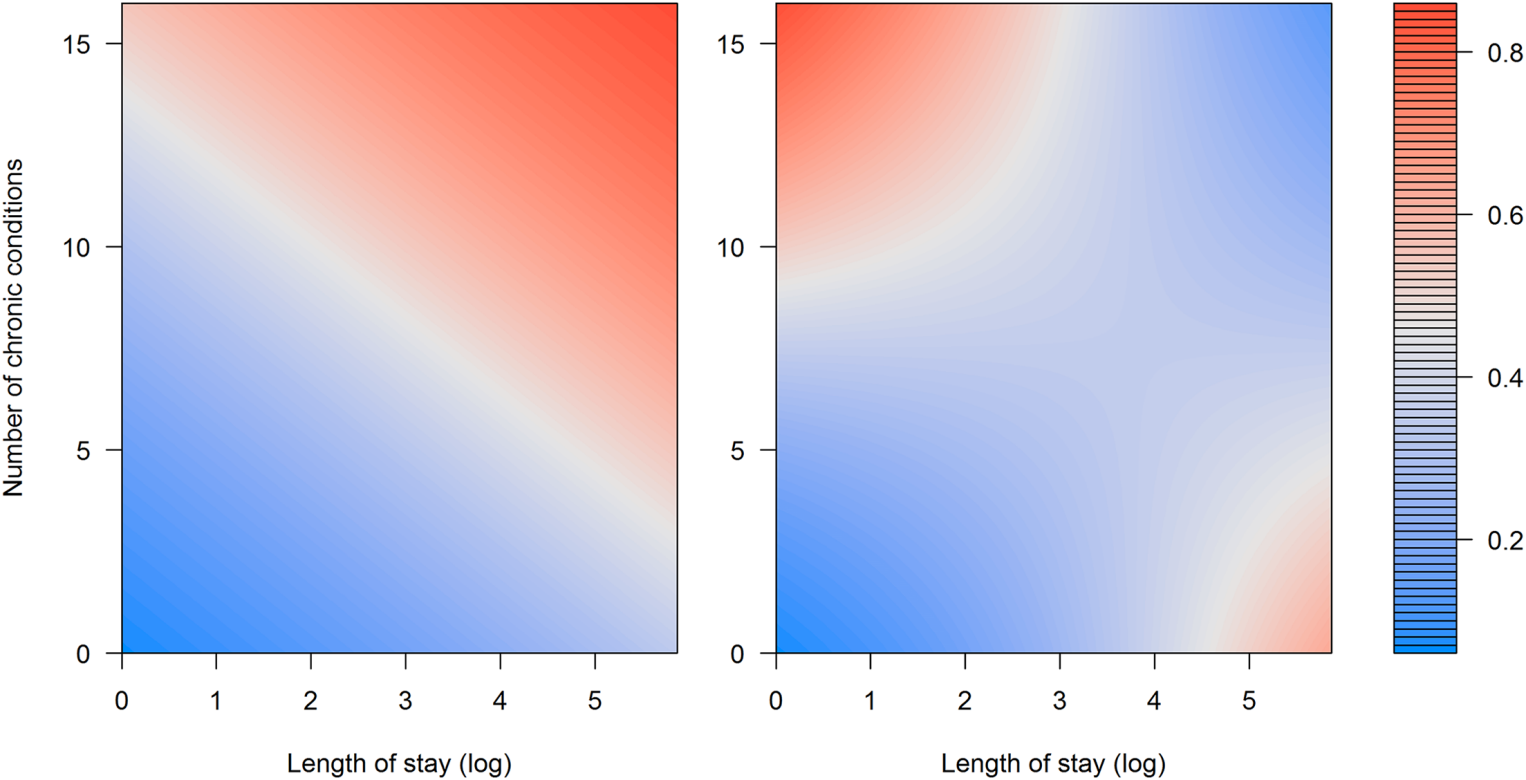
Risk of readmission with and without the interaction term. Surface plot of the response (risk of readmission) from the model without (left) and with interaction between length of stay (LOS_LOG) and number of chronic diseases (NCHRONIC).

To observe this phenomenon, also known as Simpson’s Paradox [21], we took a closer look and built two logistic regression models using only LOS_LOG and NCHRONIC with and without an interaction term. Fig 3 presents a response value of the model with interaction term (left) and without interaction term (right).

It can be seen that without the interaction term, we are not able to model the fact that by increasing LOS_LOG and NCHRONIC simultaneously, the risk of readmission actually drops. In the end, the effect of the interaction term might not be as significant as it seems for Fig 3, mainly because of the small number of patients with extremely high LOS_LOG and NCHRONIC, but this example still demonstrates the importance of interaction term inclusion in predictive modeling.

## Discussion and Conclusions

This paper presents an approach to model comorbidities by introducing additional features that can represent cases when one of the diagnoses is not present while the other is. This scenario is important in cases where a number of different but similar diagnoses are present on the medical record and we would like to exclude only one of them to define a specific subgroup of patients.

To demonstrate different possibilities in reduction of the final set of comorbidity based features, we compared the proposed approach to a variant using boosted decision trees. The proposed approaches outperformed boosted decision trees in terms of AUC and number of selected features. However, it has to be noted that it would be possible to reduce the number of selected features in decision trees without significant loss of predictive performance as we demonstrated in our earlier study [22]. On the other hand, even if we would be able to make single decision trees more comprehensible by reducing their complexity, it would still be difficult to interpret them, because of the boosting approach that results in multiple decision trees that need to be interpreted.

In contrast to a similar approach called Logic Regression [10], the proposed approach allows interactions between different types of features, although we focus this study primarily on discovery of comorbidities that can improve the predictive performance. Additionally, our approach is better suited for larger and more complex problems due to the screening phase that can significantly lower the computational complexity of the interaction discovery process. It would be possible to extend the proposed approach by inclusion of more complex interactions involving multiple features by simply re-running steps 1 and 2 of the proposed approach.

Results in this study demonstrate that in some cases, selected features represent combinations of diagnoses that would not be observed using simple interaction terms often used in regression based predictive modeling. As demonstrated with an increasing number of studies focusing on disease associations based on data from EMR [23], such interactions may lead to discovery of new knowledge. The practical value of the proposed approach reflects in improved comprehensibility of obtained predictive models by slightly improving their classification performance at the same time.

## Supporting Information

S1 File Complete list of ranked features. Four lists present the most frequent positive and negative coefficients including comorbidity terms for both proposed approaches (OPT and 1SE).
(PDF)
