# Supplementary material for: Comprehensible Predictive Modeling Using Regularized Logistic Regression and Comorbidity Based Features
Source: PLoS One. 2015 Dec 8;10(12):e0144439. doi: 10.1371/journal.pone.0144439 (PMC4672891; doi:10.1371/journal.pone.0144439)
Supplement: S1 File — Four lists present the most frequent positive and negative coefficients including comorbidity terms for both proposed approaches (OPT and 1SE). (PDF) [file pone.0144439.s001.pdf]

	Positive 1SE	Final set presence (%)
1	Int.ICD_28800=1.ICD_20400=0	100,00%
2	Int.ICD_V5811=0.ICD_1940=1	100,00%
3	Int.ICD_V5811=0.ICD_2841=1	100,00%
4	LOS_LOG	100,00%
5	NCHRONIC	100,00%
6	Int.ICD_28800=0.ICD_20400=1	99,90%
7	Int.ICD_V5811=1.ICD_28800=0	99,90%
8	Int.ICD_V5811=0.ICD_78061=1	99,29%
9	Int.ICD_V5811=1.ICD_2841=0	97,48%
10	Int.ICD_V5811=0.ICD_20400=1	96,77%
11	Int.ICD_V5811=1.ICD_20400=0	95,97%
12	Int.ICD_V5811=0.ICD_78060=1	93,75%
13	Int.ICD_V5811=1.ICD_78061=0	91,13%
14	Int.ICD_V5811=0.ICD_99931=1	88,31%
15	ICD_1940	88,00%
16	ICD_V427	83,27%
17	ICD_V5811	82,36%
18	FEMALE	73,89%
19	Int.ICD_V5811=1.ICD_78060=0	66,94%
20	Int.ICD_1940=1.FEMALE=0	65,02%
21	Int.ICD_V5811=1.ICD_99931=0	60,28%
22	Int.ICD_V5811=1.TOTCHG.NA=0	50,00%
23	NPR	38,71%
24	TOTCHG.NA	37,90%
25	Int.LOS_LOG.NPR	33,67%
26	ICD_1707	31,45%
27	ICD_78060	29,44%
28	Int.ICD_V5811=0.ICD_4019=1	17,44%
29	Int.NPR.LOS_LOG	16,63%
30	ICD_1985	15,02%
31	ICD_4019	13,71%
32	Int.ICD_V5811=0.ICD_1985=1	12,20%
33	ICD_99931	11,79%
34	ICD_20402	10,48%
35	Int.ICD_1940=0.ICD_20402=1	9,78%
36	Int.ICD_1940=0.ICD_1707=1	8,67%
37	Int.ICD_V5811=0.ICD_9962=1	8,67%
38	Int.ICD_V5811=0.ICD_V427=1	7,56%
39	Int.ICD_1940=0.ICD_V427=1	7,46%
40	Int.ICD_V5811=0.TOTCHG.NA=1	6,55%
41	Int.FEMALE=0.ICD_1940=1	6,45%
42	Int.ICD_V5811=0.ICD_20401=1	6,45%
43	Int.ICD_V5811=1.ICD_20401=0	6,45%
44	Int.ICD_V5811=1.ICD_9962=0	5,85%
45	ICD_78061	4,44%
46	Int.ICD_V5811=0.ICD_78791=1	3,93%
47	Int.FEMALE=0.ICD_1985=1	3,73%
48	Int.ICD_V5811=0.ICD_28800=1	3,53%
49	Int.ICD_V5811=1.ICD_4019=0	3,53%
50	Int.ICD_V5811=1.ICD_78791=0	3,53%
51	ICD_9962	2,72%
52	Int.ICD_78061=1.ICD_20400=0	2,42%
53	Int.ICD_V427=1.ICD_1940=0	2,42%
54	Int.ICD_V427=1.ICD_1985=0	2,22%
55	Int.ICD_V5811=0.ICD_2859=1	1,92%
56	Int.ICD_V5811=1.ICD_2859=0	1,92%
57	Int.ICD_1940=1.ICD_20402=0	1,81%
58	Int.ICD_1940=1.ICD_V427=0	1,61%
59	Int.ICD_V5811=1.ICD_1940=0	1,61%
60	Int.ICD_1940=1.ICD_1707=0	1,41%
61	Int.ICD_V427=0.ICD_1985=1	1,21%
62	Int.ICD_V5811=0.ICD_3314=1	1,11%
63	Int.ICD_1940=0.ICD_1970=1	0,91%
64	Int.ICD_20402=1.ICD_1985=0	0,91%
65	Int.ICD_78061=0.ICD_20400=1	0,81%
66	ICD_1970	0,71%
67	Int.ICD_1707=1.ICD_1985=0	0,71%
68	Int.ICD_1940=0.FEMALE=1	0,71%
69	Int.ICD_1940=1.ICD_1970=0	0,71%
70	Int.ICD_20402=0.ICD_1985=1	0,71%
71	ICD_2841	0,60%
72	ICD_5793	0,60%
73	Int.ICD_1985=0.ICD_20402=1	0,60%
74	Int.ICD_V5811=0.ICD_5793=1	0,60%
75	Int.ICD_V5811=1.ICD_3314=0	0,60%
76	Int.ICD_1707=0.ICD_1985=1	0,50%
77	Int.ICD_1985=0.ICD_1707=1	0,50%
78	Int.ICD_79902=1.ICD_53081=1	0,50%
79	ICD_3314	0,30%
80	Int.ICD_486=0.FEMALE=1	0,30%
81	Int.ICD_V427=0.ICD_1940=1	0,30%
82	ICD_78791	0,20%
83	Int.FEMALE=0.ICD_2875=1	0,20%
84	Int.FEMALE=1.ICD_27651=0	0,20%
85	Int.FEMALE=1.ICD_486=0	0,20%
86	Int.ICD_1707=0.ICD_20402=1	0,20%
87	Int.ICD_1707=1.ICD_20402=0	0,20%
88	Int.ICD_1940=0.ICD_78061=1	0,20%
89	Int.ICD_1940=0.ICD_V153=1	0,20%
90	Int.ICD_1940=1.ICD_78061=0	0,20%
91	Int.ICD_1940=1.ICD_V153=0	0,20%
92	Int.ICD_1985=0.ICD_V153=1	0,20%
93	Int.ICD_1985=1.FEMALE=0	0,20%
94	Int.ICD_1985=1.ICD_V153=0	0,20%
95	Int.ICD_V427=0.ICD_V153=1	0,20%
96	Int.ICD_V427=0.TOTCHG.NA=1	0,20%
97	Int.ICD_V427=1.ICD_V153=0	0,20%
98	Int.ICD_V427=1.TOTCHG.NA=0	0,20%
99	Int.ICD_V5811=1.ICD_1985=0	0,20%
100	ICD_99591	0,10%
101	Int.FEMALE=0.TOTCHG.NA=1	0,10%
102	Int.FEMALE=1.ICD_1985=0	0,10%
103	Int.FEMALE=1.ICD_2875=0	0,10%
104	Int.ICD_1940=0.ICD_2841=1	0,10%
105	Int.ICD_1940=1.FEMALE=1	0,10%
106	Int.ICD_1940=1.ICD_2841=0	0,10%
107	Int.ICD_1970=0.ICD_1985=1	0,10%
108	Int.ICD_1970=1.ICD_1985=0	0,10%
109	Int.ICD_1985=0.ICD_V427=1	0,10%
110	Int.ICD_1985=1.ICD_1707=0	0,10%
111	Int.ICD_1985=1.ICD_20402=0	0,10%
112	Int.ICD_20402=0.ICD_1970=1	0,10%
113	Int.ICD_20402=1.ICD_1970=0	0,10%
114	Int.ICD_28800=0.ICD_99931=1	0,10%
115	Int.ICD_28800=1.ICD_99931=0	0,10%
116	Int.ICD_53081=1.ICD_79902=1	0,10%
117	Int.ICD_78060=0.ICD_9962=1	0,10%
118	Int.ICD_78060=1.ICD_9962=0	0,10%
119	Int.ICD_78061=0.ICD_1940=1	0,10%
120	Int.ICD_78061=0.ICD_99931=1	0,10%
121	Int.ICD_78061=1.ICD_1940=0	0,10%
122	Int.ICD_78061=1.ICD_99931=0	0,10%

	Negative 1SE	Final set presence (%)
1	(Intercept)	100,00%
2	Int.ICD_486=0.ICD_32723=1	94,76%
3	ICD_486	85,38%
4	ICD_79902	61,19%
5	Int.ICD_486=0.ICD_3829=1	58,77%
6	ICD_49300	55,54%
7	Int.ICD_486=1.ICD_32723=0	51,92%
8	AGEMONTH	49,90%
9	Int.NCHRONIC.AGEMONTH	46,07%
10	ICD_3829	45,46%
11	Int.ICD_32723.NCHRONIC	32,96%
12	ICD_32723	28,23%
13	Int.ICD_79902=1.ICD_V151=0	22,58%
14	Int.ICD_79902=1.ICD_53081=0	19,46%
15	Int.ICD_486=1.ICD_3439=0	18,35%
16	Int.ICD_79902=0.ICD_V151=1	13,81%
17	Int.NCHRONIC.NPR	13,41%
18	Int.ICD_49300.NCHRONIC	12,80%
19	Int.ICD_79902=0.ICD_49300=1	12,00%
20	Int.ICD_486=0.ICD_49392=1	11,39%
21	Int.ICD_V151.NCHRONIC	10,79%
22	ICD_49392	9,58%
23	Int.ICD_486=1.ICD_3829=0	9,38%
24	ICD_49390	9,17%
25	Int.ICD_486=0.ICD_3439=1	8,97%
26	Int.ICD_3439.NCHRONIC	8,06%
27	Int.ICD_79902=0.ICD_49390=1	7,86%
28	Int.ICD_79902=1.ICD_49390=0	7,06%
29	ICD_V175	6,96%
30	Int.ICD_79902=0.ICD_32723=1	6,45%
31	Int.NPR.NCHRONIC	5,85%
32	Int.ICD_486=1.ICD_V151=0	5,65%
33	Int.ICD_486=1.ICD_53081=0	5,04%
34	Int.LOS_LOG.NCHRONIC	4,94%
35	ICD_V151	4,64%
36	Int.ICD_486=1.ICD_3159=0	4,64%
37	Int.ICD_79902=1.ICD_49300=0	4,33%
38	Int.ICD_79902=1.ICD_32723=0	4,23%
39	Int.NCHRONIC.LOS_LOG	4,03%
40	Int.AGEMONTH.TOTCHG_LOG	3,73%
41	Int.ICD_486=0.ICD_3159=1	3,73%
42	Int.ICD_486=0.ICD_V175=1	3,73%
43	TOTCHG_LOG	3,73%
44	Int.ICD_486=0.ICD_V151=1	3,13%
45	Int.ICD_79902=0.ICD_3159=1	3,02%
46	Int.ICD_53081.NCHRONIC	2,92%
47	Int.ICD_79902=1.ICD_3159=0	2,12%
48	Int.ICD_V151.NPR	2,12%
49	Int.ICD_486=0.ICD_49300=1	2,02%
50	Int.ICD_49300=0.ICD_79902=1	1,81%
51	Int.ICD_49300=1.ICD_79902=0	1,71%
52	Int.ICD_32723=1.ICD_79902=0	1,41%
53	Int.ICD_486.LOS_LOG	1,41%
54	ICD_49301	1,21%
55	Int.AGEMONTH.TOTCHG	1,11%
56	Int.ICD_79902=0.ICD_53081=1	1,11%
57	Int.ICD_486=0.ICD_79902=1	1,01%
58	Int.ICD_V151=0.ICD_79902=1	1,01%
59	Int.NCHRONIC.LOS	1,01%
60	ICD_49302	0,91%
61	Int.ICD_3159.NCHRONIC	0,91%
62	Int.ICD_32723=0.ICD_79902=1	0,81%
63	Int.ICD_486=0.ICD_49390=1	0,81%
64	Int.ICD_486=1.ICD_319=0	0,81%
65	Int.ICD_V5811=1.ICD_9962=1	0,81%
66	Int.ICD_486=0.ICD_319=1	0,71%
67	Int.ICD_79902=1.ICD_3439=0	0,71%
68	Int.ICD_486=0.ICD_49302=1	0,60%
69	Int.ICD_V151=1.ICD_79902=0	0,60%
70	Int.ICD_486=0.ICD_V440=1	0,50%
71	Int.ICD_486=1.ICD_49390=0	0,50%
72	Int.ICD_486=1.ICD_V440=0	0,50%
73	Int.ICD_49390=0.ICD_79902=1	0,50%
74	Int.ICD_79902=0.ICD_3439=1	0,50%
75	ICD_27651	0,40%
76	Int.ICD_49390=1.ICD_79902=0	0,40%
77	Int.ICD_486=1.ICD_34590=0	0,30%
78	Int.ICD_79902=0.ICD_51881=1	0,30%
79	Int.LOS_LOG.LOS	0,30%
80	Int.TOTCHG_LOG.AGEMONTH	0,30%
81	ICD_51881	0,20%
82	Int.AGEMONTH.LOS	0,20%
83	Int.ICD_319.NCHRONIC	0,20%
84	Int.ICD_486=0.ICD_7455=1	0,20%
85	Int.ICD_486=0.ICD_V4611=1	0,20%
86	Int.ICD_486=1.ICD_49392=0	0,20%
87	Int.ICD_486=1.ICD_79902=0	0,20%
88	Int.ICD_79902=1.ICD_27651=0	0,20%
89	Int.NCHRONIC.TOTCHG	0,20%
90	ICD_3159	0,10%
91	ICD_3439	0,10%
92	ICD_7455	0,10%
93	Int.FEMALE.NPR	0,10%
94	Int.ICD_486=0.ICD_07999=1	0,10%
95	Int.ICD_486=0.ICD_4659=1	0,10%
96	Int.ICD_486=0.ICD_49301=1	0,10%
97	Int.ICD_486=0.ICD_53081=1	0,10%
98	Int.ICD_486=1.ICD_07999=0	0,10%
99	Int.ICD_486=1.ICD_4659=0	0,10%
100	Int.ICD_486=1.ICD_49300=0	0,10%
101	Int.ICD_486=1.ICD_49300=1	0,10%
102	Int.ICD_486=1.ICD_7455=0	0,10%
103	Int.ICD_486=1.ICD_V4611=0	0,10%
104	Int.ICD_53081=0.ICD_79902=1	0,10%
105	Int.ICD_79902=0.ICD_07999=1	0,10%
106	Int.ICD_79902=0.ICD_27651=1	0,10%
107	Int.ICD_79902=1.ICD_07999=0	0,10%
108	Int.ICD_79902=1.ICD_51881=0	0,10%
109	Int.ICD_V5811.NPR	0,10%
110	Int.TOTCHG.NA.NCHRONIC	0,10%

	Positive OPT	Final set presence (%)
1	Int.ICD_28800=0.ICD_20400=1	100,00%
2	Int.ICD_28800=1.ICD_20400=0	100,00%
3	Int.ICD_V5811=0.ICD_1940=1	100,00%
4	Int.ICD_V5811=0.ICD_2841=1	100,00%
5	LOS_LOG	100,00%
6	NCHRONIC	100,00%
7	Int.ICD_V5811=1.ICD_28800=0	99,80%
8	Int.ICD_V5811=0.ICD_78060=1	99,19%
9	Int.ICD_V5811=0.ICD_78061=1	99,19%
10	Int.ICD_V5811=1.ICD_2841=0	98,99%
11	Int.ICD_V5811=1.ICD_20400=0	96,88%
12	Int.ICD_V5811=0.ICD_20400=1	96,77%
13	Int.ICD_V5811=1.ICD_78061=0	92,34%
14	NPR	88,81%
15	Int.ICD_V5811=0.ICD_99931=1	87,80%
16	FEMALE	87,60%
17	Int.ICD_V5811=1.ICD_78060=0	87,00%
18	ICD_V427	83,27%
19	Int.ICD_1940=1.FEMALE=0	77,72%
20	ICD_1940	68,25%
21	Int.ICD_V5811=1.ICD_99931=0	63,10%
22	Int.LOS_LOG.NPR	56,85%
23	ICD_V5811	56,55%
24	Int.ICD_V5811=1.TOTCHG.NA=0	50,71%
25	AGEMONTH	47,68%
26	Int.ICD_V5811=0.TOTCHG.NA=1	45,87%
27	TOTCHG.NA	41,94%
28	Int.NPR.LOS_LOG	32,26%
29	ICD_1707	31,25%
30	Int.ICD_V5811=1.ICD_V427=1	27,92%
31	ICD_78060	22,58%
32	ICD_99931	22,08%
33	Int.ICD_V5811=1.ICD_1940=0	20,36%
34	Int.ICD_V5811=0.ICD_4019=1	19,66%
35	Int.ICD_1940=0.FEMALE=1	19,15%
36	Int.ICD_V5811=0.ICD_V427=1	18,55%
37	ICD_1985	15,42%
38	ICD_78061	15,02%
39	ICD_4019	14,01%
40	Int.ICD_V5811=0.ICD_1985=1	12,20%
41	Int.ICD_1940=1.ICD_1707=1	11,69%
42	ICD_20402	10,48%
43	Int.ICD_79902=1.ICD_53081=1	9,68%
44	Int.ICD_1940=0.ICD_20402=1	9,17%
45	Int.FEMALE=0.ICD_1940=1	8,77%
46	Int.ICD_V5811=0.ICD_9962=1	8,67%
47	Int.ICD_79902=1.ICD_V151=1	8,57%
48	ICD_2841	7,36%
49	Int.ICD_V5811=0.ICD_28800=1	7,16%
50	Int.ICD_1940=0.ICD_V427=1	7,06%
51	Int.ICD_V5811=1.ICD_4019=0	6,96%
52	Int.ICD_V5811=0.ICD_20401=1	6,45%
53	Int.ICD_V5811=1.ICD_20401=0	6,45%
54	Int.ICD_1940=0.ICD_1707=1	5,95%
55	Int.ICD_V5811=1.ICD_9962=0	4,94%
56	Int.FEMALE=0.ICD_1985=1	4,33%
57	Int.ICD_V5811=0.ICD_78791=1	3,93%
58	Int.ICD_V5811=1.ICD_78791=0	3,83%
59	ICD_53081	3,63%
60	Int.ICD_486=1.ICD_32723=1	3,33%
61	Int.ICD_32723.NCHRONIC	3,02%
62	ICD_9962	2,92%
63	Int.FEMALE=1.ICD_1940=0	2,92%
64	ICD_28800	2,82%
65	Int.ICD_1940=1.ICD_20402=0	2,72%
66	Int.ICD_79902=1.ICD_3159=1	2,72%
67	Int.ICD_1940=1.ICD_1707=0	2,42%
68	Int.ICD_78061=1.ICD_20400=0	2,42%
69	Int.ICD_V427=1.ICD_1940=0	2,22%
70	Int.ICD_V5811=1.ICD_V427=0	2,22%
71	Int.ICD_1940=1.ICD_V427=0	2,02%
72	Int.ICD_V5811=1.ICD_1985=0	2,02%
73	Int.ICD_V5811=0.ICD_2859=1	1,92%
74	Int.ICD_V5811=1.ICD_2859=0	1,92%
75	Int.ICD_V427=1.ICD_1985=0	1,81%
76	Int.ICD_79902=1.ICD_49390=1	1,71%
77	Int.ICD_486=1.ICD_3439=1	1,21%
78	Int.ICD_V427=0.ICD_1985=1	1,11%
79	Int.ICD_V5811=0.ICD_3314=1	1,11%
80	Int.ICD_1940=0.ICD_1970=1	1,01%
81	Int.ICD_20402=1.ICD_1985=0	0,91%
82	Int.ICD_79902=1.ICD_3439=1	0,91%
83	TOTCHG_LOG	0,91%
84	Int.ICD_V5811=1.ICD_4019=1	0,81%
85	Int.ICD_1707=0.ICD_1985=1	0,71%
86	Int.ICD_1707=1.ICD_1985=0	0,71%
87	Int.ICD_78061=0.ICD_20400=1	0,71%
88	TOTCHG	0,71%
89	ICD_5793	0,60%
90	Int.FEMALE=1.ICD_1985=0	0,60%
91	Int.ICD_1940=1.ICD_1970=0	0,60%
92	Int.ICD_1985=0.ICD_20402=1	0,60%
93	Int.ICD_486.TOTCHG_LOG	0,60%
94	Int.ICD_79902=1.ICD_32723=1	0,60%
95	Int.ICD_V5811=0.ICD_5793=1	0,60%
96	Int.ICD_V5811=1.ICD_99931=1	0,60%
97	ICD_1970	0,50%
98	Int.ICD_1985=0.ICD_1707=1	0,50%
99	Int.ICD_20402=0.ICD_1985=1	0,50%
100	Int.ICD_V151=1.ICD_79902=1	0,50%
101	Int.ICD_V5811=1.ICD_3314=0	0,50%
102	Int.FEMALE=1.ICD_486=0	0,40%
103	Int.ICD_V427=0.ICD_1940=1	0,40%
104	ICD_3314	0,30%
105	Int.ICD_1940=1.FEMALE=1	0,30%
106	Int.ICD_486=0.FEMALE=1	0,30%
107	Int.ICD_486=1.ICD_319=1	0,30%
108	Int.ICD_79902=1.ICD_49300=1	0,30%
109	Int.ICD_V5811=1.FEMALE=0	0,30%
110	Int.NCHRONIC.TOTCHG_LOG	0,30%
111	ICD_78791	0,20%
112	Int.FEMALE=0.ICD_2875=1	0,20%
113	Int.FEMALE=1.ICD_27651=0	0,20%
114	Int.FEMALE=1.ICD_2875=0	0,20%
115	Int.ICD_1707=0.ICD_20402=1	0,20%
116	Int.ICD_1940=0.ICD_78061=1	0,20%
117	Int.ICD_1940=0.ICD_V153=1	0,20%
118	Int.ICD_1940=1.ICD_78061=0	0,20%
119	Int.ICD_1940=1.ICD_V153=0	0,20%
120	Int.ICD_1985=0.ICD_V153=1	0,20%
121	Int.ICD_1985=1.FEMALE=0	0,20%
122	Int.ICD_1985=1.ICD_1707=0	0,20%

	Negative OPT	Final set presence (%)
1	(Intercept)	100,00%
2	Int.NCHRONIC.AGEMONTH	100,00%
3	Int.ICD_486=0.ICD_32723=1	97,78%
4	ICD_486	82,96%
5	Int.ICD_486=1.ICD_32723=0	72,18%
6	Int.ICD_486=0.ICD_3829=1	69,66%
7	Int.NCHRONIC.NPR	66,13%
8	ICD_79902	57,66%
9	ICD_49300	53,73%
10	Int.NCHRONIC.LOS_LOG	52,62%
11	Int.LOS_LOG.NCHRONIC	47,38%
12	ICD_3829	39,42%
13	Int.ICD_486=0.ICD_79902=1	33,17%
14	Int.NPR.NCHRONIC	33,17%
15	Int.ICD_79902=0.ICD_49300=1	27,92%
16	Int.ICD_79902=0.ICD_V151=1	27,82%
17	Int.ICD_32723.NCHRONIC	27,72%
18	Int.ICD_486=1.ICD_3829=0	27,42%
19	ICD_32723	26,11%
20	Int.ICD_79902=1.ICD_V151=0	25,81%
21	Int.ICD_79902=0.ICD_53081=1	19,46%
22	Int.ICD_79902=1.ICD_53081=0	19,46%
23	Int.ICD_486=0.ICD_3439=1	18,65%
24	Int.ICD_486=1.ICD_3439=0	18,45%
25	Int.ICD_V5811=1.ICD_99931=1	16,83%
26	Int.ICD_486=1.ICD_79902=0	16,53%
27	Int.ICD_49300.NCHRONIC	16,53%
28	Int.ICD_486=0.ICD_49300=1	16,23%
29	Int.ICD_79902=0.ICD_49390=1	15,52%
30	Int.ICD_79902=1.ICD_49300=0	15,22%
31	Int.ICD_486=0.ICD_V151=1	14,52%
32	Int.ICD_3439.NCHRONIC	13,71%
33	Int.ICD_V151.NCHRONIC	12,10%
34	Int.ICD_486=0.ICD_49392=1	12,00%
35	TOTCHG_LOG	12,00%
36	Int.ICD_79902=1.ICD_49390=0	11,39%
37	Int.ICD_486=1.ICD_V151=0	11,29%
38	Int.NPR.AGEMONTH	11,19%
39	ICD_49390	10,38%
40	Int.TOTCHG.NA.NCHRONIC	10,08%
41	ICD_49392	9,58%
42	Int.ICD_79902=0.ICD_32723=1	9,38%
43	ICD_V151	8,97%
44	Int.ICD_486=1.ICD_79902=1	8,67%
45	Int.ICD_486=1.ICD_49300=1	8,37%
46	Int.ICD_53081.NCHRONIC	8,17%
47	Int.ICD_V5811.NPR	7,56%
48	ICD_V175	6,96%
49	Int.ICD_486=0.ICD_V175=1	6,75%
50	Int.ICD_79902=1.ICD_32723=0	6,65%
51	Int.ICD_V5811=1.ICD_2841=1	6,65%
52	Int.ICD_V5811=1.ICD_9962=1	5,95%
53	ICD_3439	5,24%
54	Int.ICD_486=1.ICD_53081=0	5,04%
55	Int.ICD_486=1.ICD_49300=0	4,64%
56	Int.ICD_486=0.ICD_53081=1	4,54%
57	Int.ICD_486=1.ICD_3159=0	4,54%
58	Int.ICD_486=0.ICD_3159=1	4,44%
59	Int.ICD_49300=1.ICD_79902=0	4,44%
60	Int.ICD_486.LOS_LOG	4,23%
61	Int.ICD_49300=0.ICD_79902=1	4,03%
62	Int.ICD_79902=0.ICD_3159=1	3,93%
63	Int.AGEMONTH.TOTCHG_LOG	3,83%
64	Int.AGEMONTH.NPR	3,63%
65	Int.ICD_V151.NPR	3,43%
66	ICD_53081	3,23%
67	Int.FEMALE.NPR	2,92%
68	Int.ICD_V5811=1.ICD_78061=1	2,82%
69	Int.ICD_V5811=1.ICD_28800=1	2,72%
70	Int.ICD_V5811=1.ICD_78060=1	2,62%
71	Int.ICD_79902=1.ICD_3159=0	2,22%
72	Int.ICD_32723=0.ICD_79902=1	1,81%
73	Int.ICD_32723=1.ICD_79902=0	1,81%
74	Int.ICD_486=0.ICD_49390=1	1,81%
75	Int.ICD_79902=0.ICD_3829=1	1,81%
76	AGEMONTH	1,61%
77	Int.ICD_486=1.ICD_49392=0	1,61%
78	Int.AGEMONTH.TOTCHG	1,51%
79	Int.ICD_79902=0.ICD_3439=1	1,31%
80	ICD_49301	1,21%
81	Int.ICD_486=1.ICD_49390=0	1,21%
82	Int.ICD_3159.NCHRONIC	1,11%
83	LOS	1,11%
84	Int.ICD_V151=0.ICD_79902=1	1,01%
85	Int.ICD_V151=1.ICD_79902=0	1,01%
86	ICD_49302	0,91%
87	Int.ICD_486=0.ICD_49302=1	0,91%
88	Int.ICD_79902=1.ICD_3439=0	0,91%
89	Int.ICD_V5811=1.ICD_78791=1	0,91%
90	Int.LOS_LOG.AGEMONTH	0,91%
91	Int.ICD_486=0.ICD_319=1	0,81%
92	Int.ICD_486=1.ICD_319=0	0,81%
93	Int.ICD_V5811.LOS_LOG	0,81%
94	ICD_3159	0,71%
95	Int.ICD_486=0.ICD_49301=1	0,71%
96	Int.ICD_49390=1.ICD_79902=0	0,71%
97	Int.ICD_V5811=1.ICD_4019=1	0,71%
98	Int.LOS_LOG.LOS	0,60%
99	Int.ICD_486=0.ICD_V440=1	0,50%
100	Int.ICD_486=1.ICD_V440=0	0,50%
101	Int.ICD_49390=0.ICD_79902=1	0,50%
102	Int.NCHRONIC.LOS	0,50%
103	Int.FEMALE=1.ICD_486=1	0,40%
104	Int.ICD_486=1.ICD_V151=1	0,40%
105	Int.ICD_79902=1.ICD_27651=0	0,40%
106	Int.ICD_79902=1.ICD_49300=1	0,40%
107	Int.ICD_V5811=1.ICD_3314=1	0,40%
108	NPR	0,40%
109	ICD_27651	0,30%
110	Int.AGEMONTH.LOS	0,30%
111	Int.AGEMONTH.LOS_LOG	0,30%
112	Int.ICD_1940=1.ICD_1970=1	0,30%
113	Int.ICD_34590.NCHRONIC	0,30%
114	Int.ICD_486=0.ICD_34590=1	0,30%
115	Int.ICD_486=1.FEMALE=1	0,30%
116	Int.ICD_486=1.ICD_34590=0	0,30%
117	Int.ICD_79902=0.ICD_27651=1	0,30%
118	Int.ICD_79902=0.ICD_51881=1	0,30%
119	Int.ICD_79902=1.ICD_3829=0	0,30%
120	Int.ICD_79902=1.ICD_3829=1	0,30%
121	Int.ICD_V5811=1.ICD_1940=1	0,30%
122	Int.ICD_V5811=1.ICD_2859=1	0,30%

123	Int.ICD_79902=1.ICD_3159=1	0,10%
124	Int.ICD_79902=1.ICD_V151=1	0,10%
125	Int.ICD_V5811=0.ICD_2875=1	0,10%
126	Int.ICD_V5811=0.ICD_99591=1	0,10%
127	Int.ICD_V5811=1.FEMALE=0	0,10%
128	Int.ICD_V5811=1.ICD_2875=0	0,10%

123	Int.ICD_1985=1.ICD_V153=0	0,20%
124	Int.ICD_V427=0.ICD_V153=1	0,20%
125	Int.ICD_V427=0.TOTCHG.NA=1	0,20%
126	Int.ICD_V427=1.ICD_V153=0	0,20%
127	Int.ICD_V427=1.TOTCHG.NA=0	0,20%
128	Int.TOTCHG_LOG.NCHRONIC	0,20%
129	ICD_2859	0,10%
130	ICD_3159	0,10%
131	ICD_34590	0,10%
132	ICD_99591	0,10%
133	ICD_V151	0,10%
134	Int.FEMALE=0.ICD_27651=1	0,10%
135	Int.FEMALE=1.TOTCHG.NA=0	0,10%
136	Int.ICD_1707=1.ICD_20402=0	0,10%
137	Int.ICD_1940=0.ICD_2841=1	0,10%
138	Int.ICD_1940=1.ICD_1970=1	0,10%
139	Int.ICD_1940=1.ICD_2841=0	0,10%
140	Int.ICD_1970=0.ICD_1985=1	0,10%
141	Int.ICD_1970=1.ICD_1985=0	0,10%
142	Int.ICD_1985=0.FEMALE=1	0,10%
143	Int.ICD_1985=0.ICD_V427=1	0,10%
144	Int.ICD_20402=0.ICD_1970=1	0,10%
145	Int.ICD_20402=1.ICD_1970=0	0,10%
146	Int.ICD_28800=0.ICD_99931=1	0,10%
147	Int.ICD_28800=1.ICD_99931=0	0,10%
148	Int.ICD_4019=0.ICD_1985=1	0,10%
149	Int.ICD_486=1.ICD_3159=1	0,10%
150	Int.ICD_486=1.ICD_V4611=1	0,10%
151	Int.ICD_49390=1.ICD_79902=1	0,10%
152	Int.ICD_53081=1.ICD_79902=1	0,10%
153	Int.ICD_78060=0.ICD_1970=1	0,10%
154	Int.ICD_78060=0.ICD_9962=1	0,10%
155	Int.ICD_78060=1.ICD_9962=0	0,10%
156	Int.ICD_78061=0.ICD_1940=1	0,10%
157	Int.ICD_78061=0.ICD_99931=1	0,10%
158	Int.ICD_78061=1.ICD_1940=0	0,10%
159	Int.ICD_78061=1.ICD_99931=0	0,10%
160	Int.ICD_79902=1.ICD_51881=1	0,10%
161	Int.ICD_V5811=0.ICD_2875=1	0,10%
162	Int.ICD_V5811=0.ICD_99591=1	0,10%
163	Int.ICD_V5811=1.ICD_2875=0	0,10%
164	Int.ICD_V5811=1.ICD_5793=0	0,10%
165	Int.ICD_V5811=1.ICD_78060=1	0,10%

123	Int.TOTCHG_LOG.AGEMONTH	0,30%
124	Int.FEMALE=1.ICD_27651=1	0,20%
125	Int.ICD_1940=1.FEMALE=1	0,20%
126	Int.ICD_1940=1.ICD_78061=1	0,20%
127	Int.ICD_1940=1.ICD_V153=1	0,20%
128	Int.ICD_28800=1.ICD_20400=1	0,20%
129	Int.ICD_319.NCHRONIC	0,20%
130	Int.ICD_486=0.ICD_7455=1	0,20%
131	Int.ICD_486=0.ICD_V4611=1	0,20%
132	Int.ICD_486=1.ICD_V4611=0	0,20%
133	Int.ICD_79902=1.ICD_51881=0	0,20%
134	Int.ICD_V427=1.TOTCHG.NA=1	0,20%
135	Int.NCHRONIC.TOTCHG	0,20%
136	ICD_319	0,10%
137	ICD_4659	0,10%
138	ICD_51881	0,10%
139	ICD_7455	0,10%
140	Int.FEMALE=1.TOTCHG.NA=1	0,10%
141	Int.ICD_1985=1.ICD_V153=1	0,10%
142	Int.ICD_28800=1.ICD_99931=1	0,10%
143	Int.ICD_486=0.ICD_07999=1	0,10%
144	Int.ICD_486=0.ICD_4659=1	0,10%
145	Int.ICD_486=1.ICD_07999=0	0,10%
146	Int.ICD_486=1.ICD_4659=0	0,10%
147	Int.ICD_486=1.ICD_7455=0	0,10%
148	Int.ICD_53081=0.ICD_79902=1	0,10%
149	Int.ICD_78060=1.ICD_9962=1	0,10%
150	Int.ICD_78061=1.ICD_1940=1	0,10%
151	Int.ICD_78061=1.ICD_99931=1	0,10%
152	Int.ICD_79902=0.ICD_07999=1	0,10%
153	Int.ICD_79902=1.ICD_07999=0	0,10%
154	Int.ICD_79902=1.ICD_486=0	0,10%
155	Int.ICD_V427=1.ICD_V153=1	0,10%
156	Int.ICD_V5811=1.ICD_1985=1	0,10%
157	Int.ICD_V5811=1.ICD_2875=1	0,10%
158	TOTCHG	0,10%
